# Exploring the Role of Neutrophil-Related Genes in Osteosarcoma via an Integrative Analysis of Single-Cell and Bulk Transcriptome

**DOI:** 10.3390/biomedicines12071513

**Published:** 2024-07-08

**Authors:** Jing Lu, Jiang Rui, Xiao-Yu Xu, Jun-Kang Shen

**Affiliations:** 1 Department of Radiology, The Second Affiliated Hospital of Soochow University, Suzhou 215025, China; jing1999lu@163.com; 2 Institute of Diagnostic and Interventional Radiology, Shanghai Sixth People’s Hospital Affiliated to Shanghai Jiao Tong University School of Medicine, Shanghai 200235, China; jr1127404006@163.com (J.R.); 13774230386@163.com (X.-Y.X.)

**Keywords:** osteosarcoma (OS), neutrophil-related genes (NRGs), least absolute shrinkage and selection operator (LASSO), prognostic model, tumor microenvironment (TME)

## Abstract

Background: The involvement of neutrophil-related genes (NRGs) in patients with osteosarcoma (OS) has not been adequately explored. In this study, we aimed to examine the association between NRGs and the prognosis as well as the tumor microenvironment of OS. Methods: The OS data were obtained from the TARGET-OS and GEO database. Initially, we extracted NRGs by intersecting 538 NRGs from single-cell RNA sequencing (scRNA-seq) data between aneuploid and diploid groups, as well as 161 up-regulated differentially expressed genes (DEGs) from the TARGET-OS datasets. Subsequently, we conducted Least Absolute Shrinkage and Selection Operator (Lasso) analyses to identify the hub genes for constructing the NRG-score and NRG-signature. To assess the prognostic value of the NRG signatures in OS, we performed Kaplan–Meier analysis and generated time-dependent receiver operating characteristic (ROC) curves. Gene enrichment analysis (GSEA) and gene set variation analysis (GSVA) were utilized to ascertain the presence of tumor immune microenvironments (TIMEs) and immunomodulators (IMs). Additionally, the KEGG neutrophil signaling pathway was evaluated using ssGSEA. Subsequently, PCR and IHC were conducted to validate the expression of hub genes and transcription factors (TFs) in K7M2-induced OS mice. Results: FCER1G and C3AR1 have been identified as prognostic biomarkers for overall survival. The findings indicate a significantly improved prognosis for OS patients. The effectiveness and precision of the NRG signature in prognosticating OS patients were validated through survival ROC curves and an external validation dataset. The results clearly demonstrate that patients with elevated NRG scores exhibit decreased levels of immunomodulators, stromal score, immune score, ESTIMATE score, and infiltrating immune cell populations. Furthermore, our findings substantiate the potential role of SPI1 as a transcription factor in the regulation of the two central genes involved in osteosarcoma development. Moreover, our analysis unveiled a significant correlation and activation of the KEGG neutrophil signaling pathway with FCER1G and C3AR1. Notably, PCR and IHC demonstrated a significantly higher expression of C3AR1, FCER1G, and SPI1 in Balb/c mice induced with K7M2. Conclusions: Our research emphasizes the significant contribution of neutrophils within the TIME of osteosarcoma. The newly developed NRG signature could serve as a good instrument for evaluating the prognosis and therapeutic approach for OS.

## 1. Introduction

Osteosarcoma (OS) is a common primary bone tumor that predominantly affects children, adolescents, and young adults [1,2]. It commonly manifests in the metaphysis of long bones and is characterized by a bleak prognosis and a high prevalence of functional impairment [2,3]. The 5-year overall survival rate for localized OS averages 80%, particularly when neoadjuvant chemotherapy is combined with extensive surgical resection [4,5]. However, individuals with metastatic disease exhibit significantly lower rates of both short-term and long-term survival [6,7]. Therefore, it is necessary to contribute to the enhancement of osteosarcoma treatment by identifying new targets and biomarkers.

In previous research, the focus was primarily on investigating the role of individual oncogenes, such as p53 [8], SOX2 [9], MALAT1 [10], IGF-2 [11], cyclin E1 [12], C3AR1 [13], and ATG16L1 [14], as single hub genes in osteosarcoma (OS). In comparison to past research focusing on individual oncogenes, the recent studies have identified multiple clusters of hub genes that serve as characteristic biomarkers or metastatic biomarkers for OS. These clusters are associated with various functions, including focal adhesion, type I interferon signaling, cell–cell adhesion, etc. [8,15,16,17,18,19,20,21]. Despite these advancements, the current body of research remains insufficient, and the detection of pulmonary micrometastases is often overlooked, resulting in a poor prognosis [22]. Hence, it is of significance to understand the functional implications of cancer-specific mRNAs in osteosarcoma biology.

The tumor immune microenvironment (TIME) exhibits elevated immunogenicity and infiltration of immune cells, indicating that a thorough investigation of TIME is important for the prognosis and treatment of tumors [23,24]. Consequently, conducting a comprehensive analysis of the association between TIME signatures and overall survival could provide insights into the pathogenesis of osteosarcoma.

The role of neutrophils in tumor progression has garnered significant research attention in recent years among the various types of tumor-infiltrating immune cells [25]. Neutrophils are pivotal in the immune system as they regulate immune responses, combat infections, and maintain tissue homeostasis [25,26]. Recent studies have highlighted the importance of neutrophil extracellular traps (NETs), which are neutrophil-mediated immune processes, in tumor development. These NETs play a crucial role in both innate and adaptive immune responses triggered by infectious and sterile stimuli [27].

Recent studies have demonstrated that neutrophil extracellular traps (NETs) exert a significant impact on various stages of the metastatic cascade, encompassing the advancement, infiltration, and migration of primary tumors, viability within the circulatory system, chemotactic attraction towards secondary sites, extravasation, establishment, and proliferation of metastatic tumor cells [28]. These findings elucidate the influence of the tumor microenvironment on the functional alteration of neutrophil subtypes, highlighting the crucial involvement of neutrophils in tumor development, metastasis, therapeutic response, and evasion of immune surveillance [29].

Despite the established association between neutrophils and the progression and metastasis of solid tumors [30,31,32], there is a limited number of studies that have comprehensively investigated the role of NRGs in OS. In this study, we employed machine learning techniques (including univariate Cox regression analyses, LASSO, Kaplan–Meier (KM), and time-dependent receiver operating characteristic (ROC) curves) to construct and validate risk profiles for OS patients based on NRGs. Initially, we conducted an assessment of the prognostic significance of NRGs in osteosarcoma by utilizing data from the TCGA and GEO databases. Subsequently, we devised and validated a novel signature comprising NRGs to effectively forecast the prognosis of osteosarcoma. Following this, we categorized OS patients into high- and low-risk groups and evaluated their immune microenvironments. Additionally, we conducted a thorough screening of potential TFs that could potentially regulate the two hub genes. Furthermore, we examined the expression of the two genes and TFs in K7M2-induced osteosarcoma mice.

## 2. Materials and Methods

### 2.1. Database Selection

The scRNA-seq data GSE162454, consisting of 6 osteosarcoma tissues, were obtained from the GEO platform (https://www.ncbi.nlm.nih.gov/geo/) (accessed on 3 March 2023), utilizing 10X Genomics technology [33]. The TARGET database (https://ocg.cancer.gov/programs/target) (accessed on 5 March 2023) provided the Therapeutically Applicable Research to Generate Effective Treatment-Osteosarcoma (TARGET-OS) dataset. The TARGET-OS dataset comprises 88 samples, each with comprehensive survival data and corresponding expression profiles. Additionally, the GSE21257 datasets were acquired from the GEO database (https://www.ncbi.nlm.nih.gov/geo/) (accessed on 3 March 2023). The RNA-seq data from GSE21257 encompassed 34 metastatic and 19 non-metastatic patients, all of which have associated survival information and expression profiles (Table S1). Detailed clinical information for the 88 samples in the TARGET database and the 53 samples in the GSE21257 dataset is provided separately in Supplementary Tables S2 and S3, respectively.

This study employed a publicly accessible dataset that had previously obtained ethical approval. Informed consent was obtained from all participants, and the study was conducted in accordance with the principles delineated in the Declaration of Helsinki.

### 2.2. Single-Cell Sequencing Analysis

The analysis of the single-cell data involved the utilization of the “Seurat” package, which facilitated the identification of highly variable genes, centralization, principal component analysis (PCA) downscaling, cell clustering, t-distributed stochastic neighbor embedding (t-SNE), uniform manifold approximation and projection (UMAP) nonlinear downscaling, differential gene identification, and cell annotation. Additionally, the “copycat” package in R was employed to predict the copy number variation (CNV) of each cell, thereby enabling the inference of diploid (normal cells) and aneuploid (tumor cells) [34].

Subsequently, we acquired a set of 50 hallmark pathways from the Molecular Signatures Database (MsigDB, http://www.gsea-msigdb.org/gsea/index.jsp) (accessed on 8 March 2023) through a curated selection process [35]. Additionally, we obtained 31 curated tumor microenvironment (TME) signatures [36]. By conducting ssGSEA analysis on both tumor cells and normal cells within each sample, we derived the enrichment score for each pathway. To visualize the differences in pathway enrichment scores and TME signatures between diploid (normal cells) and aneuploid (tumor cells), we employed boxplots and heatmaps. Furthermore, we employed the DoRothEA R package to analyze the transcription factors across OS clusters.

### 2.3. Screening of Neutrophil-Associated DEmRNAs

The mRNA expression data and clinical information of patients were obtained by downloading from each dataset. Based on the follow-up data, the patients were categorized into metastatic and non-metastatic groups. The identification of differentially expressed mRNAs (DEmRNAs) between the two groups was performed using the Linear Models for Microarray Analysis (limma) package in R software. Moreover, genes exhibiting adjusted *p*-values or *p*-values of <0.05 along with |log2FC| ≥ 1.5 were identified as statistically different genes. The visualization of the DEmRNAs was achieved by generating a heatmap using the “pheatmap” package in R software. Finally, the DEmRNAs associated with neutrophils were identified by finding the intersection between the DEmRNAs and genes related to neutrophils.

### 2.4. Functional Enrichment Analysis for scRNA seq

Conducted a Gene Ontology (GO) and Kyoto Encyclopedia of Genes and Genomes (KEGG) analysis of the differential marker genes between subclusters using ClusterProfiler 4.0 in R [37]. We used the GSVA package [38] to analyze the differential marker genes among subclusters. All gene sets were obtained from the Molecular Signatures Database MSigDB (https://www.gsea-msigdb.org/gsea/msigdb/index.jsp) (accessed on 8 March 2023) [35]. We used the Scillus package to perform Gene Set Enrichment Analysis (GSEA) on the differential marker gene expression among subclusters (https://github.com/xmc811/Scillus) (accessed on 10 March 2023).

### 2.5. Construction and Evaluation of a Neutrophil-Related Prognostic Signature

Univariate Cox regression analyses and LASSO were employed to identify the most significant candidate genes for the development of a prognostic signature. The risk score for each patient was computed using a specified formula: Risk score = Σin(Coefi × Xi) [3]. To evaluate the prognostic signature’s accuracy, Kaplan–Meier (KM) and time-dependent receiver operating characteristic (ROC) curves were generated in the training cohort. Furthermore, the association between risk scores and various clinical characteristics was investigated. Ultimately, the prognostic signature’s accuracy was validated in an independent validation cohort.

### 2.6. Establishment and Validation of a Predictive Nomogram

To improve the clinical applicability of the established prognostic signature, risk scores and clinical characteristics were integrated to establish a nomogram to predict the 3- and 5-year survival rate of patients with osteosarcoma. C-index, ROC, and DCA curves were used to assess the accuracy of the predictive nomogram.

### 2.7. Analysis of TME, Immunomodulators and Signal Pathways

In this study, first, 31 functional gene expression signatures (Fges) [36] were analyzed by one-sample gene set enrichment analysis (ssGSEA) performed with R’s “gsva” package. Furthermore, immunomodulators [39], including antigen presentation, cell adhesion, co-inhibitor co-stimulator, ligand, receptor, and other, were estimated based on the hub genes. Lastly, the enrichment of 29 signal pathways [40] in the three datasets was analyzed using the hub genes. 

### 2.8. Cell Culture

The murine osteosarcoma cell line K7M2 was acquired from the Cell Bank of the Chinese Academy of Sciences (Shanghai, China). The cells were cultured in a humidified incubator at 37 °C with 5% CO_2_, using a culture medium consisting of DMEM supplemented with 10% (*v*/*v*) FBS and 1% P&S [41].

### 2.9. Osteosarcoma Mouse Model Construction

A total of 10 Balb/c mice, aged 3–4 weeks and obtained from Gempharmacy Co., Ltd., Nanjing, China, were individually housed within the specific pathogen-free barrier system (SPF) at the experimental animal center of Shanghai Sixth People’s Hospital, affiliated with Shanghai Jiao Tong University School of Medicine (Ethical approval number: 2020–0221). Osteosarcoma tumors were induced in the mice by injecting 1 × 10^6^ K7M2 cells into the left thigh muscle using 100 µL of Hanks Balanced Salt Solution [41]. After 28 days from the cell injection, the mice were euthanized for analysis through CO_2_ asphyxiation. The freshly obtained osteosarcoma (OS) tissues were collected for subsequent analysis using Western blotting (WB) and real-time quantitative polymerase chain reaction (qPCR). Simultaneously, the OS tissues were embedded in paraffin, sectioned into slides, and subjected to hematoxylin and eosin (HE) staining as well as immunohistochemical (IHC) staining.

### 2.10. Quantitative Real-Time PCR (RT-qPCR)

Total RNA extraction from cells was performed according to the manufacturer’s instructions using a TRIzol reagent (Invitrogen, Carlsbad, CA, USA). Reverse transcription of cDNA from RNA was then performed using PrimeScript RT Master Mix (Takara). The primer sequences for the tissues of the thoracic and abdominal arteries were as follows (5′-3′): GAPDH (forward: 5′-CCTCGTCCCGTAGACAAAATG-3′, reverse: 5′-TGAGGTC-AATGAAGGGGTCGT-3), C3AR1 (forward: 5′-GGAACTGTGGGCTCATTGCT-3′, reverse: 5′-CAACTTCCCTTTTGATTGTCTTCTC-3′), IGFBP3 (forward: 5′-CCAAGCGTGAGACAGAATAC-3′, reverse: 5′-AGGAGGGATAGGAGCA-AGAT-3′), SPI1 (forward: 5′-TTTGAGAACTTCCCTGAGAACCAC-3′, reverse: 5′-GCATGTAGGAAACCTGGTGACTG-3′) synthesized by Shanghai Generay Biotech. The primer sequence was downloaded from Primer Bank (https://pga.mgh.harvard.edu/primerbank/index.html) (accessed on 15 March 2023) and synthesized by Shanghai Sangon Biotechnology (Shanghai, China). The GAPDH gene was applied as an internal reference. Target gene expression was determined using the 2^−ΔΔ^Ct method.

### 2.11. Immunohistochemistry (IHC)

The tissues were preserved with 4% paraformaldehyde (15 min), immersed in paraffin, and cut into an average of 4 μm slices. The antigens were extracted after dewaxing and dehydration. The completed slices were then fixed with 3% hydrogen peroxide for 20 min and blocked at room temperature for 15 min with 5% BSA. Whereafter, anti-C3AR1 (bs-2955R 1:200; Bioss), anti-FCER1G (bs13167R 1:200; Bioss), and anti-SPI1 (66618-2-lg 1:1000; Proteintech) were incubated at 4 °C overnight using antibody diluent solution (Life-iLab, Shanghai, China). The segments were colored by the color-developing agent for 3–15 min and then were washed, redyed, dehydrated, transparent, and sealed in sequence, which were detected by SP kits (Solarbio, Beijing, China). Finally, these slices were put under a light microscope for observation and photography.

### 2.12. Statistical Analysis

Bioinformatics analyses and R packages were all conducted by R software (version 4.3.1). The means between two groups of normally distributed variables were compared by Student’s *t*-test. The Wilcoxon test was used to compare data that were not normally distributed. Univariate Cox regression analyses and LASSO were employed to identify the most significant candidate genes. Kaplan–Meier (KM) analysis, ROC curves, and AUC values were utilized to construct and validate the prognostic signatures in the training set, test set, and validation set. Furthermore, * *p* < 0.05, ** *p* < 0.01, and *** *p* < 0.001 were considered significant.

## 3. Results

### 3.1. ScRNA Sequencing Data Analysis

The workflow of the study design is depicted in Figure S1. ScRNA-seq data (10X Genomics) from GEO162454 encompassing 6 osteosarcoma tissues (four men and two women, 13–45 years old) were obtained (Table S4). A total of 47,245 cells were captured following initial single-cell data filtration, and 29,509 cells were selected for subsequent analysis after quality control (Table S5).

**Figure 1:**
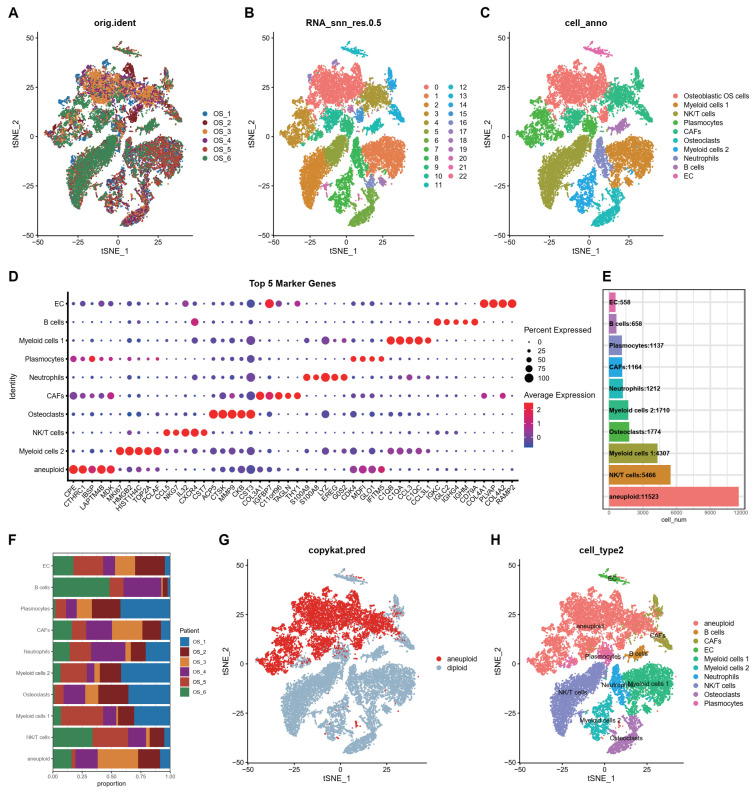
scRNA-Seq analysis for GSE162454. (**A**) The plot generated using T-distributed stochastic neighbor embedding (tSNE) technique is colored by patients. (**B**) The t-SNE plot is colored based on the 23 different cell types. (**C**) The tSNE plot was colored to display 10 distinct cell types. (**D**) The heatmap illustrates the top five markers of the ten clusters. (**E**,**F**) The boxplot displays the cell numbers and proportion of the ten cell clusters. (**G**,**H**) The t-SNE plot is colored based on the aneuploid and diploid groups (**G**), as well as the 10 distinct cell types (**H**), utilizing the copykat R package for CNV analysis.

After applying batch effects and batch normalization using the Harmony R package, a total of twenty-three distinct cell clusters with unique gene expression patterns were obtained at a resolution of 0.5 (Figure 1A,B). Following annotation, these clusters were consolidated into 10 cell clusters, namely osteoblastic OS cells, B cells, cancer-associated fibroblasts (CAFs), endothelial cells (EC), myeloid cells 1, myeloid cells 2, neutrophils, natural killer/T cells (NK/T cells), osteoclasts, and plasmocytes (Figure 1C–F). These annotated clusters align with the findings of the original study [33]. To emphasize the significance of the neutrophil cluster, we isolated it from the myeloid cell 1 cluster. The Seurat package’s function FindAllMarkers was utilized to identify differentially expressed genes (DEGs) across all cell clusters (Table S6). Additionally, ScRNA-seq data were analyzed for copy number variations (CNV) using the copykat R package, resulting in the classification of clusters into aneuploid and diploid groups (Figure 1G,H). FindMarkers was then employed to identify DEGs between these two groups, resulting in the identification of 2806 DEGs (Table S7). From Table S6, 770 neutrophil DEGs were extracted and intersected with Table S7, resulting in the identification of 538 neutrophil-related genes (NRGs) (Table S8).

**Figure 2:**
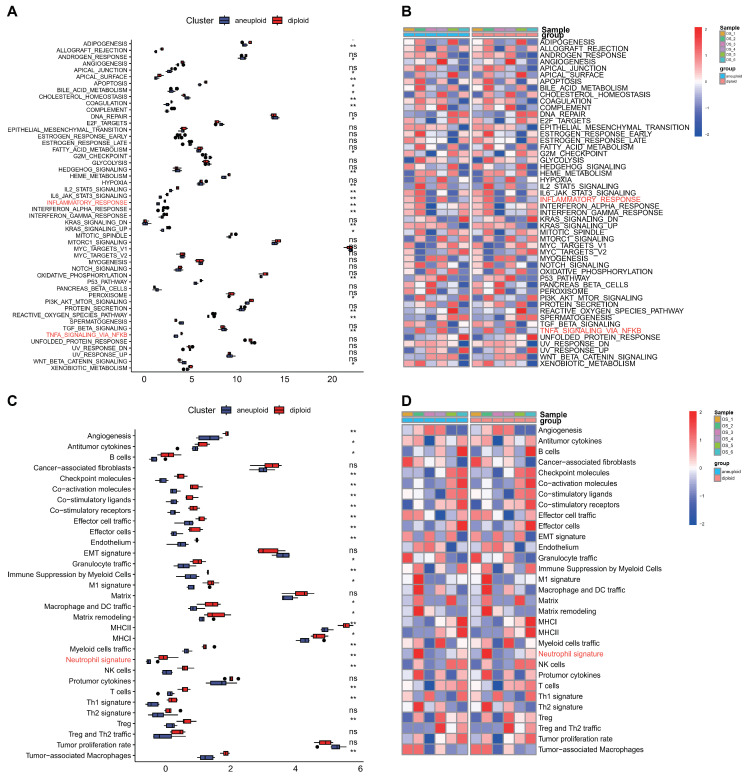
HALLMARK_genesets and functional gene expression signatures (Fges) analysis for GSE162454 between aneuploid and diploid groups. (**A**,**B**) Comparison of the disparities in the 50 HALLMARK_genesets between aneuploid and diploid groups in the GSE162454 sets. (**C**,**D**) Comparison of the variations in the Fges between aneuploid and diploid groups in the GSE162454 datasets, with red indicating case group and dark blue representing control groups. ns: *p* > 0.05; * *p* < 0.05, ** *p* < 0.01.

After differentiating tumor cells from OS tissues, the ssGSEA method was utilized to calculate the enrichment scores of 50 HALLMARK_genesets and functional gene expression signatures (Fges) in single cells, comparing aneuploid and diploid groups. Our results demonstrated that the scores of 50 HALLMARK_genesets and Fges in tumor cells were significantly lower than those in normal cells, particularly in relation to the inflammatory pathway or neutrophil signature (indicated by the red character). This suggests that tumors may employ a strategy of inhibiting inflammatory-related processes in order to protect themselves and ensure survival (see Figure 2A–D).

### 3.2. Hub Genes Detection from scRNA-Seq and TARGET-OS

**Figure 3:**
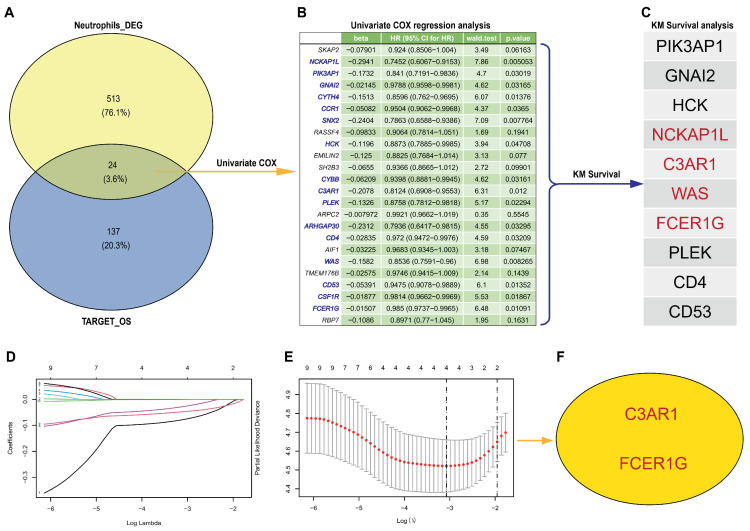
The hub genes detection form NRGs and DEGs from Target-OS. (**A**) The Venn diagram illustrates the presence of 24 differentially expressed genes (DEGs) resulting from the overlap between 538 neutrophil-related genes (NRGs) and 161 up-regulated DEG mRNAs from Target-OS. (**B**) The most significant candidate genes were identified through the implementation of univariate Cox regression analyses. (**C**) Prognostic signature genes were selected based on their association with survival outcomes, as demonstrated by Kaplan–Meier (KM) survival curves in Target-OS. (**D**,**E**) The LASSO algorithm was employed to select hub genes. (**F**) The LASSO algorithm identified two genes, namely C3AR1 and FCER1G, as significant.

In order to determine the most effective prognostic signature, we conducted both univariate Cox analysis and LASSO Cox regression analysis on prognosis-related ANRGs. First, the Target-OS dataset, consisting of 56 metastatic and 22 non-metastatic osteosarcoma patients, was utilized for the selection of biomarkers. A total of 238 down-regulated and 161 up-regulated DEG mRNAs associated with osteosarcoma metastasis were identified. By intersecting these with 538 neutrophil-related genes (NRGs) and the 161 up-regulated DEG mRNAs, we were able to identify 24 neutrophil-associated mRNAs (Figure 3A).

Subsequently, univariate Cox regression analyses were conducted to identify the most statistically significant candidate genes, resulting in the identification of 16 genes (Figure 3B, *p* < 0.05). Then, we assessed the prognostic value of these 16 genes using Kaplan–Meier survival curves and found that ten genes (NCKAP1L, PIK3AP1, GNAI2, HCK, C3AR1, PLEK, CD4, WAS, CD53, and FCER1G) were significantly associated with patient prognosis (*p* < 0.05) in Target-OS (Figure 3C). Furthermore, LASSO Cox regression analysis was employed to further reduce dimensionality and construct a gene signature, resulting in the identification of 4 biomarkers (NCKAP1L, C3AR1, WAS, FCER1G) at lambda.min (Figure 3D,E). Finally, we validated the prognostic significance of these four genes in GEO (GSE21257) datasets and found that only two genes (C3AR1 and FCER1G) were significantly associated with patient prognosis (Figure 3F, *p* < 0.05). Thus, the two biomarkers were selected as the final diagnostic prediction biomarkers.

### 3.3. Establish an Effective Prognostic Risk Model

In order to develop a prognostic risk model, the TARGET-OS cohort was divided into training sets (*n* = 50) and testing sets (*n* = 33) at a ratio of 6:4, while the GEO cohort (*n* = 53) was used for validation purposes after removing 5 cases with unknown survival time (*n* = 2) and zero survival time (*n* = 3). Subsequently, the training set was utilized to construct the final prognostic model, represented by the equation Risk Score = −0.0812 × C3AR1 − 0.012 × FCER1G. 

**Figure 4:**
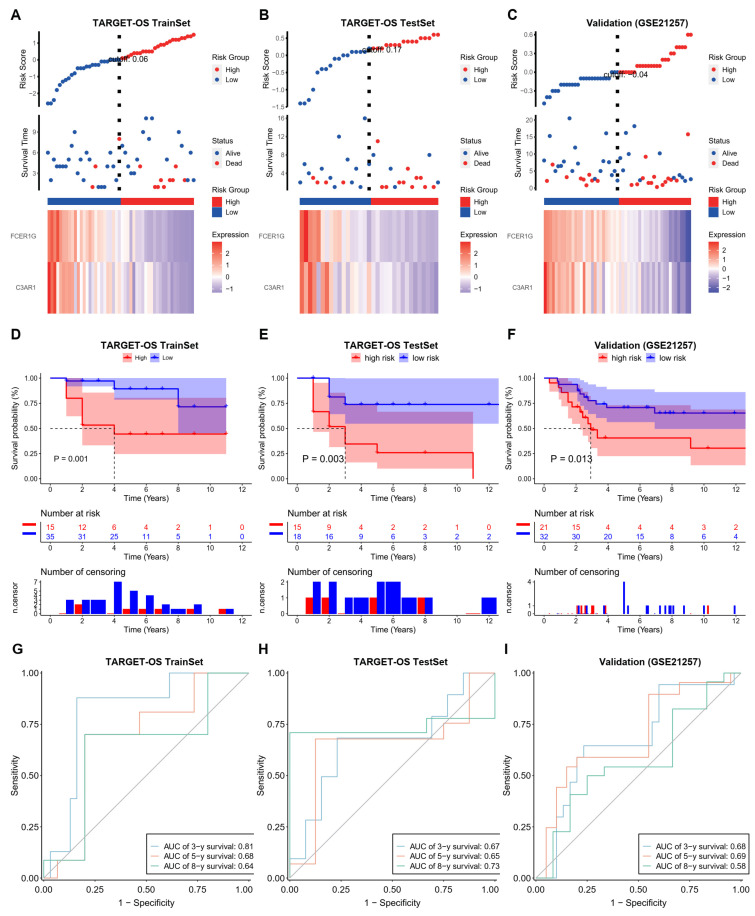
The training, test and validation of the prognostic power of NRG score in OS. (**A**–**C**) The distribution of risk scores, patient statuses, and mRNA expression levels of hub genes are depicted in a heatmap for the TARGET-OS training, TARGET-OS test, and validation cohorts (GSE21257) (**D**–**F**) The Kaplan-Meier survival curve is analyzed for the TARGET-OS training, TARGET-OS test, and validation cohorts (GSE21257). (**G**–**I**) The AUC values for the TARGET-OS training, TAR-GET-OS test, and validation cohorts (GSE21257) are assessed at 3, 5, and 8 years.

The Kaplan–Meier survival curves demonstrated significant differences in survival rates among the three cohorts, which were stratified based on the risk score (Log-rank, *p* < 0.05). This suggests that the risk score has the potential to serve as a reliable predictor of patient prognosis (Figure 4A–C). The time-dependent receiver operating characteristic (ROC) curve was utilized to assess the prognostic capability of the risk scoring model, and the area under the curve (AUC) values for the training set, test set, and validation set at 3-year, 5-year, and 8-year were all found to be greater than 0.6, indicating a robust predictive ability of the prognostic risk model for the survival of patients with TARGET-OS (Figure 4D–F). Furthermore, a nomogram was constructed based on the prognostic signature (Figure 4G–I). The calibration curves for the 3-year, 5-year, and 8-year survival demonstrated a substantial overlap between the observed survival rate and the predicted survival rate derived from the nomogram. This observation suggests that the nomogram exhibits a good predictive value.

### 3.4. Establishment and Validation of Nomogram

**Figure 5:**
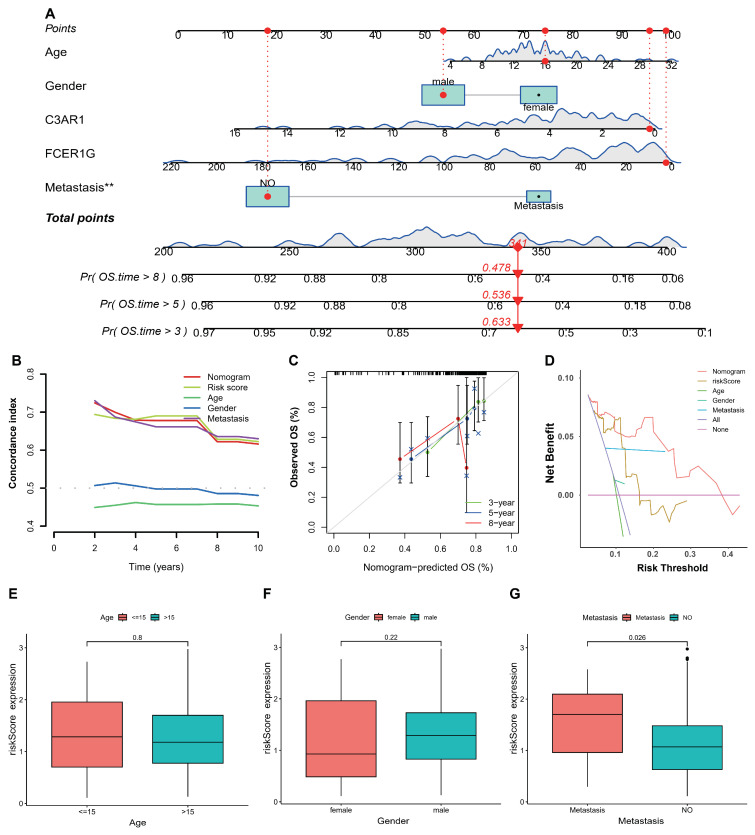
Establishment of the nomogram in TARGET-OS. (**A**) The nomogram predicted the 3- 5- and 8-year survival risk in osteosarcoma patients. (**B**) the DCA also revealed that the nomogram had high potential clinical utility. (**C**) Calibration curves of the nomogram for predicting the probability of OS at 3-, 5-, or 8-year. (**D**) DCA also revealed that the nomogram had high potential clinical utility. (**E**–**G**) Boxplots of the relationship between riskscore and clinical characteristics. ** *p* < 0.01.

The nomogram served as a robust instrument for the comprehensive assessment of patient prognosis by incorporating clinical variables. In the present investigation, the construction of the nomogram was carried out using the TARGET-OS databases, with the aim of predicting the survival rates at 3, 5, and 8 years for individuals diagnosed with osteosarcoma. The inclusion of gender, age, metastasis status, and two key genes facilitated the development of the nomogram for osteosarcoma. The graphical representation in Figure 5A depicts the nomograms for predicting the survival outcomes of patients with TARGET-OS at 3, 5, and 8 years. The results of the study demonstrated that the nomogram, riskscore, and metastasis exhibited a high concordance index with time, as depicted in Figure 5B. Additionally, the calibration plots indicated that the performance of the nomogram was comparable to that of the good model, as shown in Figure 5C. Furthermore, the decision curve analysis (DCA) revealed that the nomogram possessed significant potential for clinical utility, as depicted in Figure 5D. Lastly, the study investigated the correlations between clinical features and the riskscore, particularly in relation to patients’ metastasis status in TARGET-OS, as illustrated in Figure 5E–G. 

### 3.5. C3AR1 and FCER1G Reshapes the TME and Immune Signal Pathways of OS

**Figure 6:**
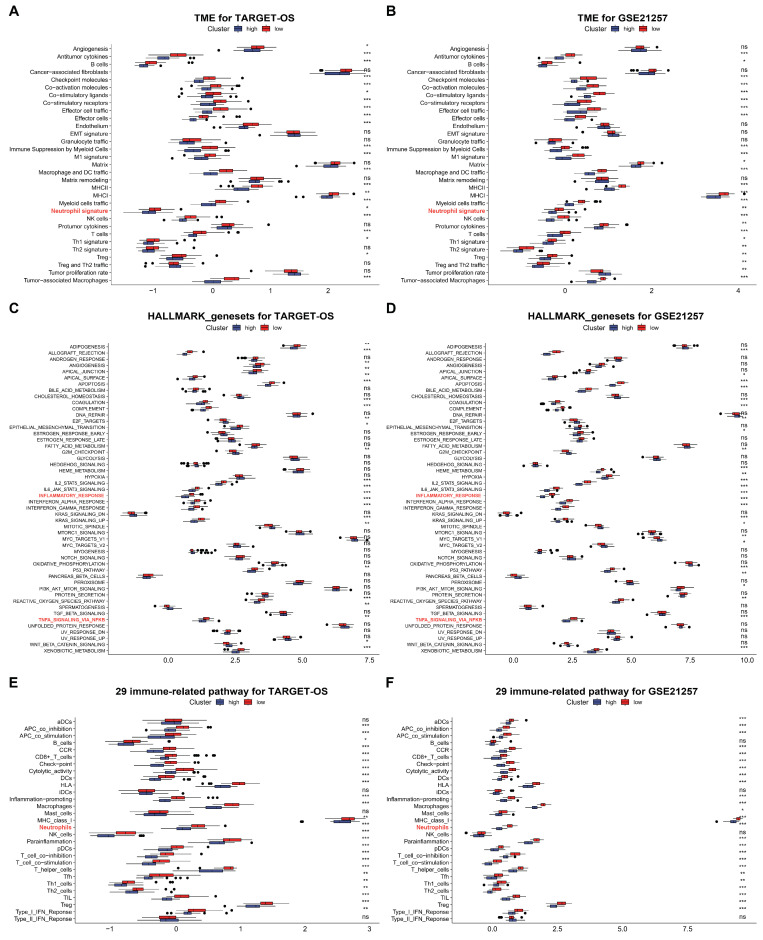
The hub genes remodel TME in OS. (**A**,**B**) Comparison of the differences in the Fges of TME between high and low risk groups in TARGET-OS and GSE21257 cohorts, respectively. (**C**,**D**) Comparison of the differences in the 50 HALLMARK_genesets between high and low risk groups in TARGET-OS and GSE21257 cohorts, respectively. (**E**,**F**) Comparison of the differences in the 29 immune related pathways between high and low risk groups in TARGET-OS and GSE21257 cohorts, respectively. Red indicates low risk groups, while dark blue indicates high risk groups. ns: *p* > 0.05; * *p* < 0.05, ** *p* < 0.01, *** *p* < 0.001.

Based on the significant enrichment of C3AR1 and FCER1G in osteosarcoma in both single-cell RNA sequencing experiments, we conducted an analysis of the immune components of the functional gene expression signatures (Fges), specifically focusing on 50 HALLMARK_genesets. To perform this analysis, we utilized data from the TARGET-OS cohort and GEO datasets (GSE21257). From the Fges signatures, we selected 31 to investigate the TIME in this study. Our analysis revealed that, based on the results presented in Figure 6A,B, 89.7% (22 out of 31) and 89.7% (25 out of 31) of the Fges signatures exhibited significant differences between high and low risk groups in the TARGET-OS and GSE21257 cohorts, respectively.

In comparison to low-risk groups, our analysis revealed that high-risk groups in the TARGET-OS and GSE21257 cohorts exhibited a higher enrichment of HALLMARK signal pathways, with 48% (24/50) and 52% (26/50) pathways enriched, respectively (Figure 6C,D). Additionally, we observed that the majority of immune-related pathways were enriched in high-risk groups as compared to low-risk groups in both cohorts (Figure 6E,F). These findings suggest that the subgroup with high-risk levels in OS tissue displayed a greater abundance of TIME and immune signal pathways.

### 3.6. ESTIMATE Evaluation and Immunomodulators Analysis Based on the Two Hub Genes

**Figure 7:**
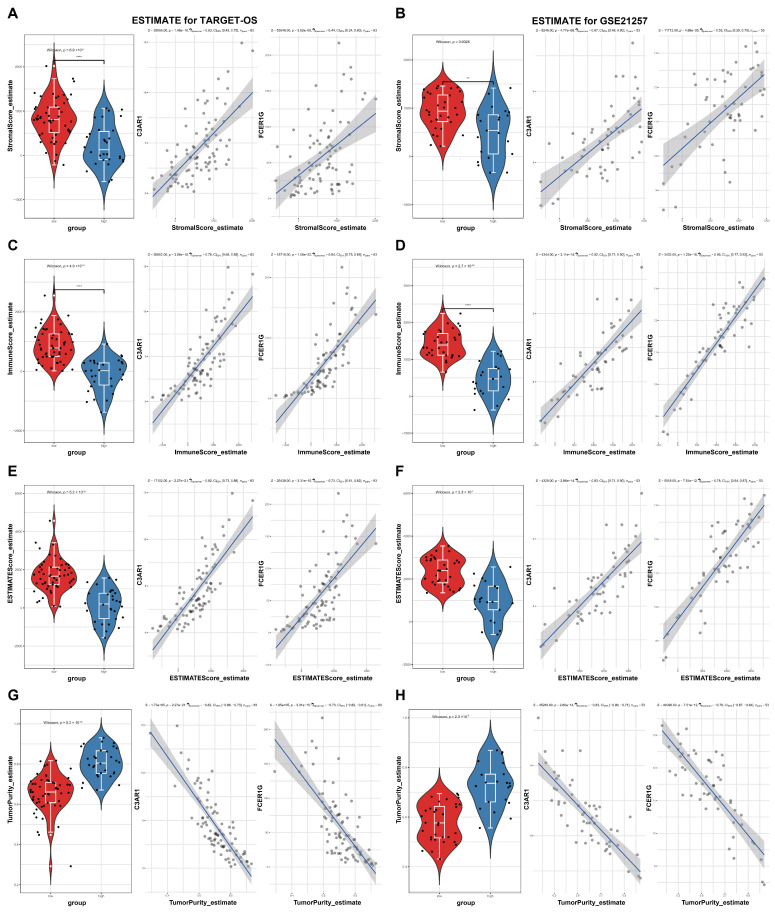
ESTIMATE evaluation based on the two hub genes. (**A**–**H**) In both the TARGET-OS and GSE21257 cohorts, a comparison was made between high and low samples regarding the stromal scores, immune scores, ESTIMATE scores, and tumor purity. Additionally, it was observed that C3AR1 or FCER1G exhibited a significant positive correlation with stromal scores, ESTIMATE scores, and immune scores, while displaying a strong negative correlation with tumor purity in both cohorts. ** *p* < 0.01, ****: *p* < 0.0001.

**Figure 8:**
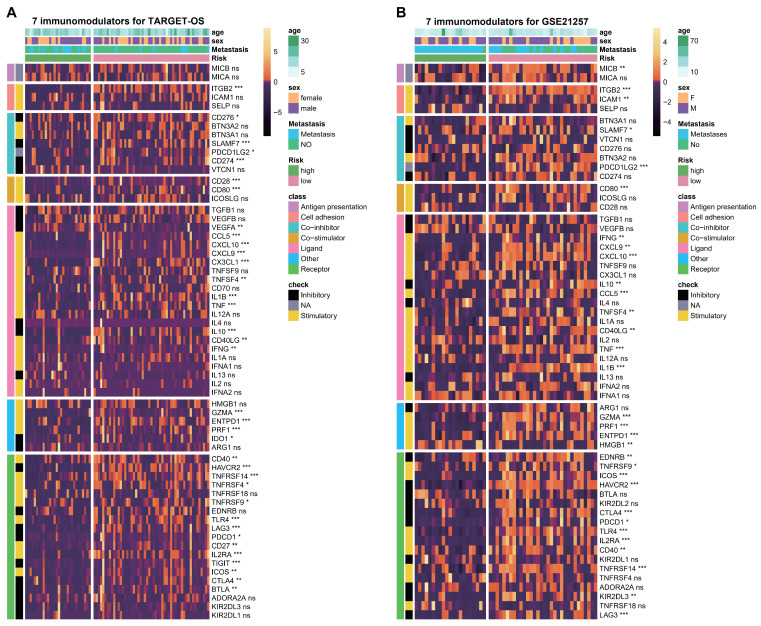
Evaluation of immunomodulators based on the two hub genes. (**A**,**B**) Heatmap displaying enrichment of immunomodulators through 7 algorithms between high- and low groups according to C3AR1 and FCER1G levels in the TARGET-OS (**A**) and GSE21257 cohorts (**B**). ns: *p* > 0.05; * *p* < 0.05, ** *p* < 0.01, *** *p* < 0.001.

It has been observed that OS tissue in a high-risk group exhibits an increased abundance of TIME and immune signal pathways. Subsequently, an investigation was conducted on the ESTIMATE scores and immunomodulators using the C3AR1 and FCER1G hub genes for the two datasets. The results, as illustrated in Figure 7A–H, demonstrate significant differences in stromal scores, ESTIMATE scores, immune scores, and tumor purity between high and low samples based on the C3AR1 and FCER1G genes within the two cohorts (*p* < 0.05). Simultaneously, a significant positive correlation was observed between C3AR1 or FCER1G and the stromal scores, ESTIMATE scores, and immune scores, while a substantial negative correlation was noted with tumor purity in both cohorts (Figure 7A–H). Furthermore, when comparing high samples to low samples, more than half of the high overall survival (OS) samples demonstrated significantly reduced levels of immunomodulators in both the TARGET-OS and GSE21257 cohorts (Figure 8A,B). These findings suggest that individuals with elevated levels of C3AR1 and FCER1G in their OS tissue exhibit a decreased abundance ofTIME and immunomodulators.

### 3.7. Correlation between FCER1G and C3AR1 and KEGG Neutrophil Signaling Pathway in OS

**Figure 9:**
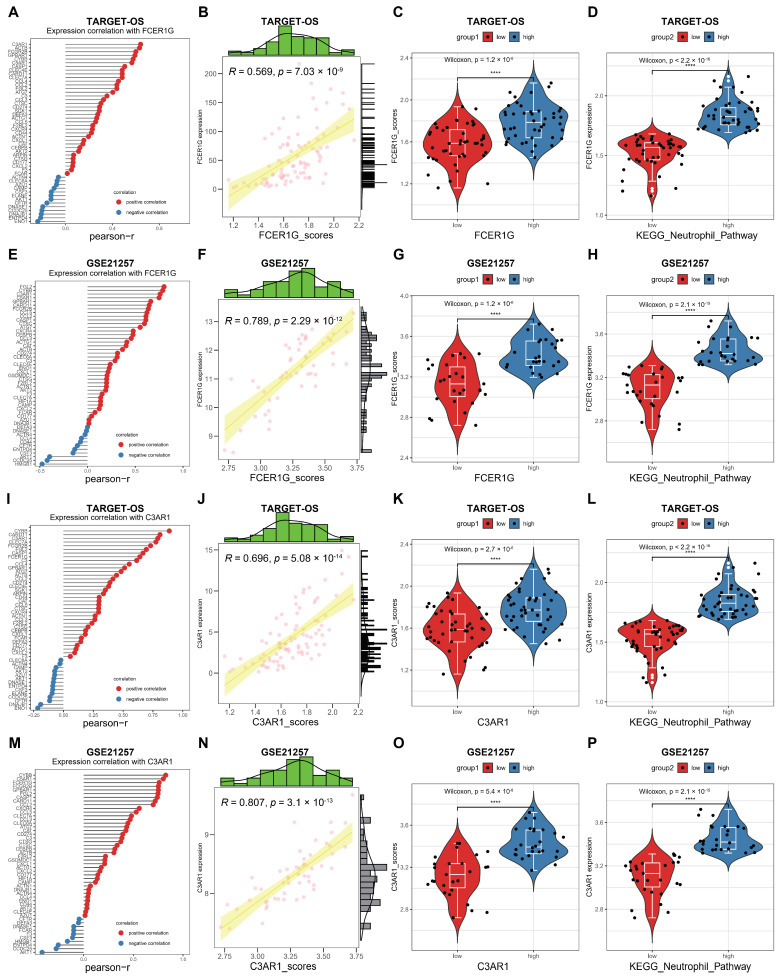
FCER1G/C3AR1 correlated KEGG NSP in OS. (**A**) The correlation between FCER1G and KEGG NSP genes was examined in the TARGET-OS dataset. (**B**) The correlation between FCER1G and KEGG NSP scores was investigated in the TARGET-OS dataset. (**C**) A comparison was made between the KEGG NSP scores in subgroups with high and low FCER1G expression in the TARGET-OS dataset. (**D**) The expression of FCER1G was compared between subgroups with high and low KEGG NSP scores in the TARGET-OS dataset. (**E**) The correlation between FCER1G and KEGG NSP genes was examined in the GSE21257 dataset. (**F**) The correlation between FCER1G and KEGG NSP scores was investigated in the GSE21257 dataset. (**G**) A comparison was made between the KEGG NSP scores in subgroups with high and low FCER1G expression in the GSE21257 dataset. (**H**) The expression of FCER1G was compared between subgroups with high and low KEGG NSP scores in the GSE21257 dataset. (**I**) The correlation between C3AR1 and KEGG NSP genes was examined in the TARGET-OS dataset. (**J**) The correlation between C3AR1 and KEGG NSP scores was investigated in the TARGET-OS dataset. (**K**) A comparison was made between the KEGG NSP scores in subgroups with high and low C3AR1 expression in the TARGET-OS dataset. (**L**) The expression of C3AR1 was compared between subgroups with high and low KEGG NSP scores in the TARGET-OS dataset. (**M**) The correlation between C3AR1 and KEGG NSP genes was examined in the GSE21257 dataset. (**N**) The correlation between C3AR1 and KEGG NSP scores was investigated in the GSE21257 dataset. (**O**) A comparison was made between the KEGG NSP scores in subgroups with high and low C3AR1 expression in the GSE21257 dataset. (**P**) The expression of C3AR1 was compared between subgroups with high and low KEGG NSP scores in the GSE21257 dataset. ****: *p* < 0.0001.

The activation of the KEGG neutrophil signaling pathway (NSP) by C3AR1 and FCER1G in OS was investigated. Subsequently, a correlation analysis was conducted between FCER1G/C3AR1 and the KEGG NSP using two datasets. Notably, our analysis revealed a significant positive correlation between FCER1G and the majority of genes within this pathway (Figure 9A,E). Moreover, the ssGSEA algorithm was utilized to assign scores to the KEGG NSP in the two datasets. Based on the obtained scores, a distinct positive correlation was observed between FCER1G and the KEGG NSP (Figure 9B,F). Lastly, the current study provides evidence of the capacity to differentiate pathway scores among subgroups characterized by high and low FCER1G expression (Figure 9C,G), as well as to discern FCER1G expression levels between subgroups with high and low pathway scores (Figure 9D,H). Similarly, a comparable positive association between C3AR1 and the KEGG NSP was observed in OS (Figure 9I–P). These findings suggest that elevated FCER1G/C3AR1 expression is implicated in the progression of AP through involvement in the KEGG NSP.

### 3.8. Screening the Key Transcription Factors (TFs) Regulating the Two Hub Genes

**Figure 10:**
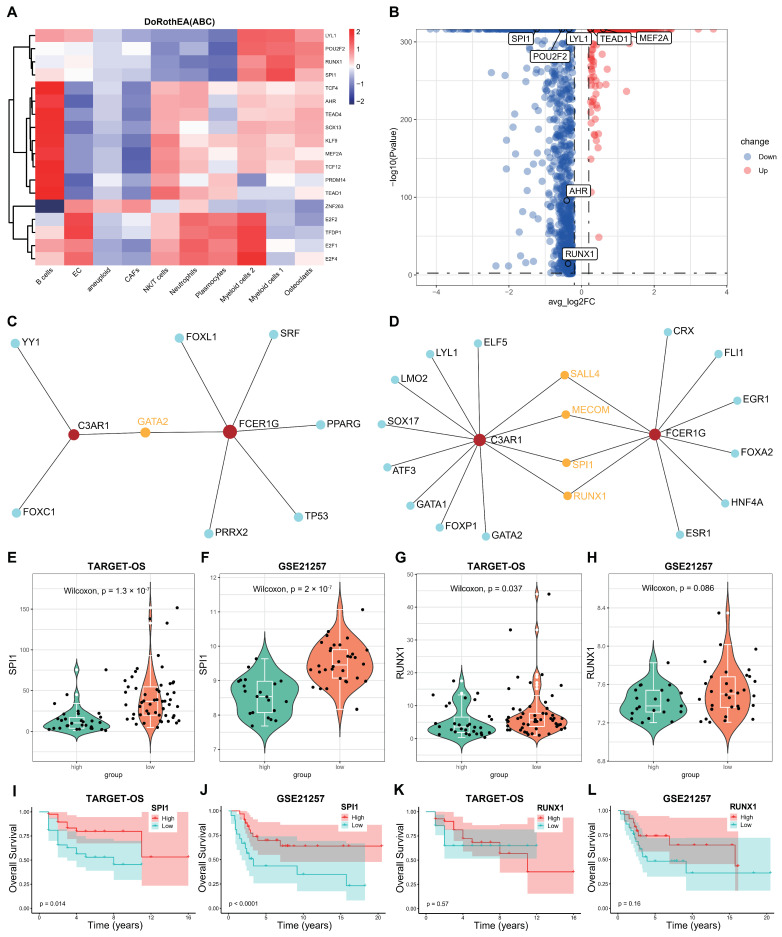
SPI1 was identified as a potential key TF in OS. (**A**) The heatmap illustrates the identification of 18 transcription factors (TFs) using the DoRothEA R package in scRNA-seq data. (**B**) The volcano plot generated from the analysis of scRNA-seq data reveals the presence of eight differentially expressed genes (DEGs) that exhibit significant differences between aneuploid and diploid groups. (**C**,**D**) The identification of potential transcription factors (TFs) that may regulate the C3AR1 and FCER1G genes involves screening two databases (JASPAR and ChEA) through the utilization of NetworkAnalyst 3.0. (**E**–**H**) The expression levels of SPI1 and RUNX1 were found to be decreased in both the TARGET-OS and GSE21257 datasets. (**I**–**L**) The Kaplan–Meier survival plots demonstrate the overall survival (OS) of the SPI1 and RUNX1 genes in both the TARGET-OS and GSE21257 datasets.

To further elucidate the underlying mechanism of OS, we initially conducted an analysis of the active transcription factors (TFs) in OS using the DoRothEA R package in single-cell RNA sequencing (scRNA-seq). We identified a total of 18 active TFs across the 10 clusters (Figure 10A), with only eight TFs exhibiting significant differences between the aneuploid and diploid groups (Figure 10B). Subsequently, we employed NetworkAnalyst 3.0 to screen for potential TFs that could regulate the two crosstalk genes, C3AR1 and FCER1G, utilizing data from three databases (ENCODE, JASPAR, and ChEA). Figure 10C,D displays the transcription factors (TFs) that may regulate the two crosstalk genes, obtained from the JASPAR and ChEA databases, labeled as 1 and 4, respectively. By integrating TFs from the single-cell RNA sequencing (scRNA-seq) data, we identified SPI1 and RUNX1 as the shared TFs in both the scRNA-seq and NetworkAnalyst datasets. Subsequently, we computed the mRNA expression levels (Figure 10E–H) and Kaplan–Meier survival (Figure 10I–L) of these two TFs in the TARGET-OS and GSE21257 cohorts. Notably, the expression levels of SPI1 exhibited significant differences across both datasets. Therefore, it can be inferred that SPI1 may serve as the pivotal transcription factor responsible for regulating the interplay between the two genes in osteosarcoma. These findings suggest that SPI1 facilitates the proliferation of osteosarcoma cells by upregulating the expression of C3AR1 and FCER1G, as well as activating the neutrophil signaling pathway outlined in the KEGG database.

### 3.9. In Vivo Analyses

**Figure 11:**
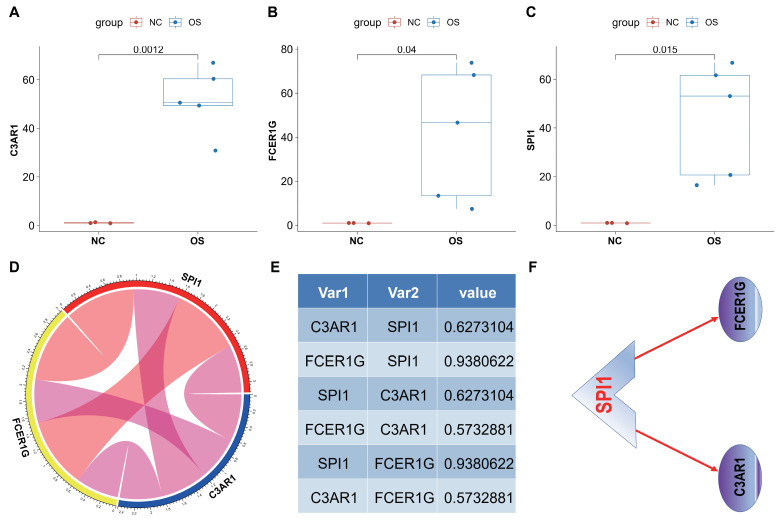
Evaluation of C3AR1, FCER1G and SPI1 in OS animal model. (**A**–**C**) The three genes (C3AR1 (**A**), FCER1G (**B**) and SPI1 (**C**)) showed significant upregulation in OS mice tissue samples compared to normal tissue samples. (**D**,**E**) Correlation circle pipes and light image show the correlations among C3AR1, FCER1G and SPI1 genes in OS mice tissues. (**F**) The potential transcription factor regulatory network in OS was investigated. Data were represented as mean with SD.

In order to further substantiate the expression of the C3AR1, FCER1G, and SPI1 genes, we conducted an evaluation of these genes in three K7M2-induced Balb/c mice. Initially, we conducted a comparative analysis of gene expression levels in the left thigh muscle of healthy control mice and OS tissues of K7M2-induced Balb/c mice using RT-qPCR. Our findings from RT-qPCR analysis of OS tissue samples revealed a significant upregulation of the three genes in OS tissues compared to normal tissues (Figure 11A–C). Additionally, the correlation circle pipes (Figure 11D) and cor value (Figure 11E) demonstrated positive correlations among the three genes in OS tissues. Consequently, it can be inferred that SPI1 positively regulates the two hub genes, suggesting a potential mechanism (Figure 11F).

**Figure 12:**
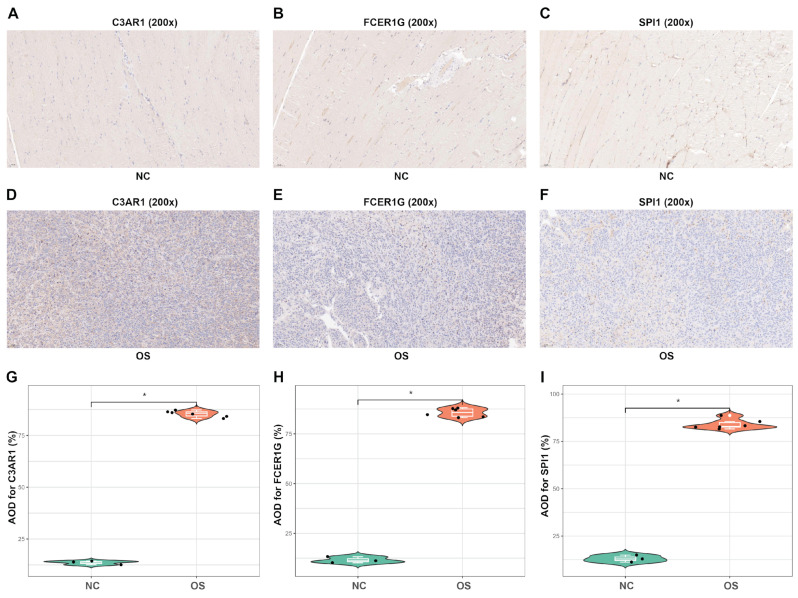
IHC for C3AR1, FCER1G and SPI1. (**A**–**C**) IHC for C3AR1, FCER1G, and SPI1 in the normal tissue samples (20× magnification). (**D**–**F**) IHC for C3AR1, FCER1G and SPI1 in the OS mice tissues (20× magnification). (**G**–**I**) The violin plots exhibit higher levels of staining for C3AR1, FCER1G and SPI1 protein expression in OS tissues as compared to normal tissue samples. AOD = Average optical density. * *p* < 0.05.

In order to assess the internal index in OS tissues, immunohistochemical staining was conducted on tissue sections to evaluate the expression of three proteins (Figure 12A–F). The results indicated that OS tissues exhibited significantly higher levels of staining for C3AR1, FCER1G, and SPI1 protein expression in comparison to normal tissues (Figure 12G–I). These findings suggest that C3AR1 and FCER1G have the potential to serve as valuable diagnostic biomarkers for OS, while SPI1 may play a crucial role as a transcription factor in regulating C3AR1 and FCER1G during the pathological progression of OS.

## 4. Discussion

Osteosarcoma (OS) is the predominant malignant bone tumor in both adult and pediatric populations, known for its pronounced inclination towards invasion and metastasis. Currently, numerous therapeutic interventions have been implemented for OS patients, encompassing surgical procedures, radiotherapy, chemotherapy, and neoadjuvant chemotherapy [42]. Nevertheless, the notable hallmark of OS is its elevated incidence of distant metastasis, which is closely associated with alterations in the expression of multiple genes and significantly influences patient prognosis [43,44]. The advancement of scRNA-seq technology has led to the identification of an increasing number of cell subclusters. This has enabled researchers to conduct in-depth investigations into heterogeneous tumors, gain insights into the tumor microenvironment, and explore the functional characteristics of novel cell populations [45].

In this study, we conducted single-cell RNA analysis to identify alterations in osteosarcoma (OS) cells. We isolated 23 major cell populations in OS tissue and annotated 10 cell types, including the neutrophil cell type, by extracting characteristic genes. Subsequently, we categorized these clusters into aneuploid and diploid groups based on copy number variation using the copykat R package. We identified 538 neutrophil-related genes (NRGs) through the intersection of 538 NRGs from scRNA-seq with 161 up-regulated differentially expressed genes (DEGs) from TARGET-OS datasets. Finally, we employed LASSO analysis to identify two hub genes, which were used to construct the NRG-score and NRG-signature.

In the context of cancer, the presence of tumor-associated neutrophils (TANs) within the tumor microenvironment (TME) has been recognized as a significant factor. TANs are capable of exerting two distinct functions within this environment. On one hand, they contribute to the promotion of tumor growth by facilitating processes such as angiogenesis, extracellular matrix remodeling, metastasis, and immunosuppression. On the other hand, neutrophils can also play a role in mounting antitumor responses through direct elimination of tumor cells and involvement in cellular networks that confer resistance against tumor development. The diverse and adaptable nature of neutrophils underlies their dual potential as TANs within the TME [25]. The infiltration of tumor-associated neutrophils (TANs) has been acknowledged as an adverse prognostic indicator in the majority of solid malignancies [46]. Within this investigation, we have identified C3AR1 and FCER1G as noteworthy core genes linked to overall survival (OS). Additionally, the Kaplan–Meier survival analysis has revealed that the risk score effectively discriminates between OS patients with favorable and unfavorable prognoses. Moreover, the time-dependent receiver operating characteristic (ROC) curves have demonstrated the high predictive accuracy of the risk score in forecasting the clinical outcomes of OS, as validated in both internal and external cohorts. Lastly, in comparison to the OS tissues observed in the normal control group, the expression levels of C3AR1 and FCER1G were notably elevated in K7M2-induced Balb/c mice. Our findings provide confirmation that NRGs serve as an adverse prognostic indicator in OS, and tumor cells may safeguard themselves by suppressing NRG activities.

Previous research studies have documented various polygenic risk-prognosis signatures that can be utilized to predict the prognosis of osteosarcoma. For instance, Lei et al. developed a 12-gene ferroptosis signature, which demonstrated AUC values of 0.818 and 0.838 at 3 and 5 years, respectively [3]. Similarly, Zhang et al. reported a 6-gene pyroptosis signature, exhibiting AUC values of 0.738 and 0.742 at 3 and 5 years, respectively [47]. Fu et al. devised a 2-gene apoptosis signature, yielding AUC values of 0.74 and 0.71 at 3 and 5 years, respectively [48]. Additionally, Qian et al. constructed a 12-gene autophagy signature, displaying AUC values of 0.814 and 0.865 at 3 and 5 years, respectively [49]. This study found that the NRGs signature displayed a high level of accuracy in prognosticating the outcome of osteosarcoma, as evidenced by AUC values of 0.81 and 0.68 at 3 and 5 years, respectively. Furthermore, a nomogram was developed that effectively combined the risk score and clinical characteristics, resulting in a robust predictive capability for the survival of osteosarcoma patients. Overall, these findings offer clinicians a valuable tool for prognosticating the prognosis of osteosarcoma patients.

Two candidate genes within the NRGs signature have been identified as having relevance to the prognosis and progression of osteosarcoma (OS). Specifically, C3AR1, a protein coding gene, encodes the protein C3a Receptor 1, which functions as an orphan G protein-coupled receptor for C3a. C3a is a proinflammatory mediator released during activation of the complement system [50], which plays a critical role in innate immunity [51]. Previous studies have demonstrated the involvement of C3AR1 mRNA in various diseases, including multiple myeloma [52], colon cancer [53], and melanoma [54]. Furthermore, C3AR1 has been closely associated with immune responses, such as sepsis [55] and Alzheimer’s disease (AD) [56]. FCER1G, also referred to as Fc receptor-gamma (FcRγ), plays a significant role in allergic reactions [57]. Recent research has demonstrated the involvement of FCER1G in the pathogenesis of various diseases, including glioma [58], diabetic kidney disease [59], endometrial cancer [60], COVID-19 [61], and osteosarcoma [62]. Our findings indicate that the mRNA expression of C3AR1 and FCER1G may serve as a predictive factor for the prognosis of osteosarcoma patients and is closely associated with metastatic progression. In summary, C3AR1 and FCER1G mRNA hold potential as prognostic markers for both prognosis and metastasis in osteosarcoma.

Moreover, the Spi-1 proto-oncogene (SPI1), which encodes PU.1 (a member of the E26-transformation-specific transcription factor family), has been found to function as an oncogene specifically activated in acute murine erythroleukemias induced by the Friend spleen focus-forming virus (SFFV) [63]. As a transcription factor, SPI1 can be recruited by the small nucleolar RNA host gene 16 to regulate downstream gene expression, thereby promoting the biological behavior of cancer cells [64]. SPI1 has been identified to facilitate the progression of multiple malignant cancers, such as melanoma [65], lung cancer [66], glioma 8 [67] and other types of neoplasia. In this study, we revealed a significant association between SPI1 mRNA levels and the prognosis of osteosarcoma patients, indicating its potential as a predictive factor. Moreover, we observed a strong correlation between SPI1 mRNA expression and the progression of metastasis in these patients.

This study has certain limitations that need to be addressed. The NRG signature was constructed and validated using a retrospective analysis of a public database; therefore, further prospective studies are necessary to assess its clinical practicability. The expression of the two genes in the NRG signature was examined in mice, but the study lacks biological experiments, necessitating additional wet experiments to investigate the function of the relevant genes. Lastly, a more comprehensive investigation is required to elucidate the molecular mechanisms through which C3AR1 and FCER1G contribute to the malignant progression of OS.

Through multiple analyses, we have determined the significance of C3AR1 and FCER1G in osteosarcoma (OS). Consequently, we have formulated a prognostic signature consisting of two genes, which enables the prediction of prognostic risk in OS patients. Notably, C3AR1 and FCER1G exhibit promising potential as prognostic biomarkers, exerting their influence on the TIME and immunoregulation. Therefore, evaluating the expression levels of C3AR1 and FCER1G holds promise as a method to identify patients who may derive benefits from immunotherapy.

## Data Availability

The datasets generated or analyzed in this study are available in open access databases. In this study we used the following databases for analysis, data acquisition and visualization: GEO (https://www.ncbi.nlm.nih.gov/geo/, accessed on 3 March 2023), the TARGET database (https://ocg.cancer.gov/programs/target, accessed on 5 March 2023).The authors declare that all relevant data supporting the findings presented in this study are available within the article and its Supplementary Information files, or, from the corresponding author upon reasonable request.

## Supplementary Materials

The following supporting information can be downloaded at: https://www.mdpi.com/article/10.3390/biomedicines12071513/s1, Figure S1: Workflow of the study; Table S1: The summary of the TARGET-OS and GEO21257 datasets; Table S2: Clinical information for TARGET-OS; Table S3: Clinical information for GSE21257; Table S4: 47,245 cells from six patients related to GSE162454 were analyzed in this study; Table S5: 29,509 cells from six patients related to GSE162454 were analyzed after initial quality control in this study; Table S6: DEGs obtained by Function FindAllMarkers; Table S7: 2806 different markers between aneuploid and diploidgroups; Table S8: 538 neutrophil-related genes.
