# Supplementary material for: Exploring the Role of Neutrophil-Related Genes in Osteosarcoma via an Integrative Analysis of Single-Cell and Bulk Transcriptome
Source: Biomedicines. 2024 Jul 8;12(7):1513. doi: 10.3390/biomedicines12071513 (PMC11274533; doi:10.3390/biomedicines12071513)
Supplement: Supplementary file 1 [file biomedicines-12-01513-s001.zip › Supplementary Materials.pdf]

Supplementary Materials for

Exploring the role of neutrophil-related genes in osteosarcoma via an integrative analysis of single-cell and bulk transcriptome

Jing Lu[1,2], M.D.; Jiang Rui [2], M.D.; Xiao-Yu Xu[2], M.D.; Jun-Kang Shen[1], M.D., Ph.D.

Institutional Affiliation

1. Department of Radiology, The Second Affiliated Hospital of Soochow University

2. Institute of Diagnostic and Interventional Radiology, Shanghai Sixth People's Hospital Affiliated to Shanghai Jiao Tong University School of Medicine

Corresponding author: Jun-Kang Shen

Address: No. 1055 Sanxiang Road, Suzhou, 215000, Jiangsu, China.

E-Mail: shenjunkang@suda.edu.cn

CONTACT: Jun-Kang Shen, **E-Mail:** shenjunkang21@163.com, **Institutional Affiliation:** Department of Radiology, The Second Affiliated Hospital of Soochow University, **Address:** No. 1055 Sanxiang Road, Suzhou, 215000, Jiangsu, China.

This file includes:

Figure S1

Tables S1, S4, S5

Supplementary Figure

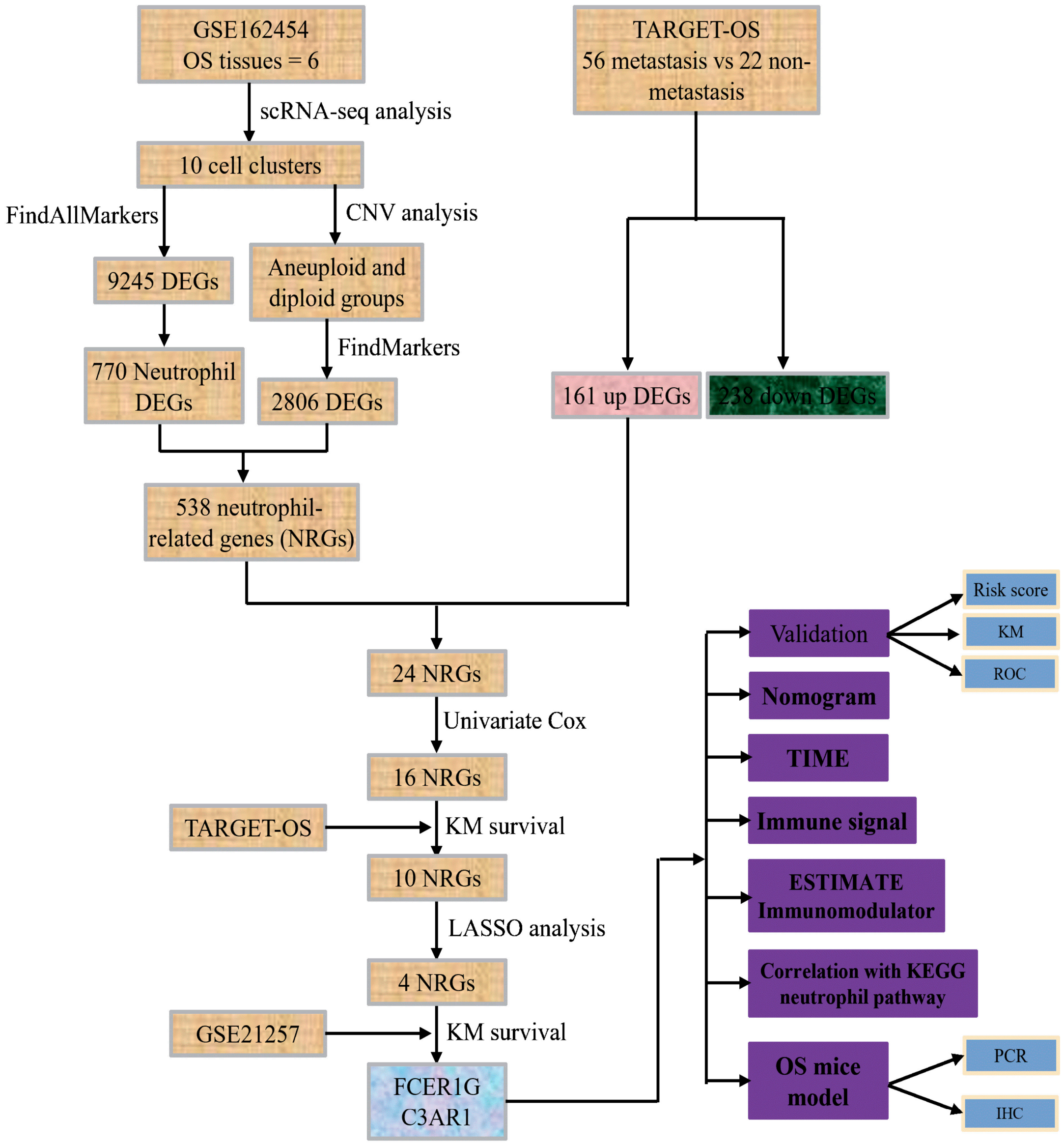

Figure S1. Workflow of the study.

Note: OS = Osteosarcoma; **CNV** = Copy number variation; **DEG** = Differentially expressed genes; NRG = Neutrophil-related genes; **TF** = Transcription factor; **KM** = Kaplan-Meier; **TIME** = Tumor immune microenvironments; **ROC** = Receiver operating characteristic; **PCR** = Polymerase chain reaction; **IHC** = Immunohistochemistry.

Supplementary Tables.

Table S1. The summary of the TARGET-OS and GEO21257 datasets.

GEO ID	Platform	Tissue Type	Sample size	Experiment type
TARGET-OS	GDC	human osteosarcoma tissues	88	RNA-seq
GSE21257	GPL10295	human osteosarcoma tissues	53	Array

Table S4. 47,245 cells from six patients related to GSE162454 were analyzed in this study.

ID	Cell counts	Percentage (%)
OS_1	7771	16.45
OS_2	7715	16.33
OS_3	9096	19.25
OS_4	4083	8.64
OS_5	9939	21.04
OS_6	8641	18.29

Note: OS = osteosarcoma

Table S5. 29,509 cells from six patients related to GSE162454 were analyzed after initial quality control in this study.

ID	Cell counts	Percentage (%)
OS_1	5505	18.66
OS_2	4502	15.26
OS_3	6486	21.98
OS_4	2642	8.95
OS_5	4185	14.18
OS_6	6189	20.97

Note: OS = osteosarcoma
